# Supplementary material for: Transcriptomic Insights into Mechanisms of Early Seed Maturation in the Garden Pea (Pisum sativum L.)
Source: Cells. 2020 Mar 23;9(3):779. doi: 10.3390/cells9030779 (PMC7140803; doi:10.3390/cells9030779)

Sprint−2(20) vs Zhewai

Global Summary

%DE = 0.82

genes with fdr < 0.2 = 1661 (764 + / 897 −)

genes with fdr < 0.1 = 1495 (679 + / 816 −)

genes with fdr < 0.05 = 1318 (586 + / 732 −)

genes with fdr < 0.01 = 895 (365 + / 530 −)

genes in genesets = 0

<FC> = 0

<t−score> = −2.65

<p−value> = 0.01

<fdr> = 0.18

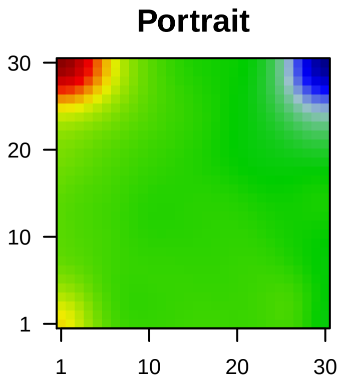

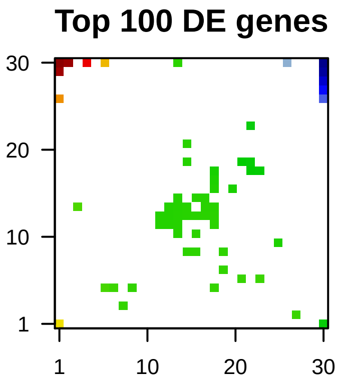

Global Genelist

Rank	ID	log(FC)	p−value	fdr Metagene	Description
1	TRINITY_DN2	−3.11	7e−11	0.00000031 x 30	
2	TRINITY_DN1	−0.04	8e−11	0.00000015 x 19	
3	TRINITY_DN8	−1.56	8e−11	0.00000037 x 1	
4	TRINITY_DN2	−0.04	3e−10	0.00000018 x 18	
5	TRINITY_DN9	−0.01	5e−10	0.00000043 x 12	
6	TRINITY_DN1	0.01	2e−09	0.00000045 x 21	
7	TRINITY_DN2	−0.03	5e−09	0.00000023 x 16	
8	TRINITY_DN1	−0.01	6e−09	0.00000043 x 13	
9	TRINITY_DN3	−0.01	6e−09	0.00000043 x 14	
10	TRINITY_DN2	−0.01	8e−09	0.00000046 x 13	
11	TRINITY_DN5	−0.01	9e−09	0.00000046 x 9	
12	TRINITY_DN1	−0.01	9e−09	0.00000043 x 12	
13	TRINITY_DN1	−0.01	9e−09	0.00000043 x 11	
14	TRINITY_DN2	−0.01	1e−08	0.00000083 x 14	
15	TRINITY_DN1	−0.01	2e−08	0.00000083 x 12	
16	TRINITY_DN1	−0.09	2e−08	0.00000082 x 18	
17	TRINITY_DN1	−0.01	2e−08	0.00000084 x 13	
18	TRINITY_DN1	−0.01	2e−08	0.00000083 x 12	
19	TRINITY_DN1	−0.01	2e−08	0.00000083 x 12	
20	TRINITY_DN1	3.45	2e−08	0.00000081 x 30	

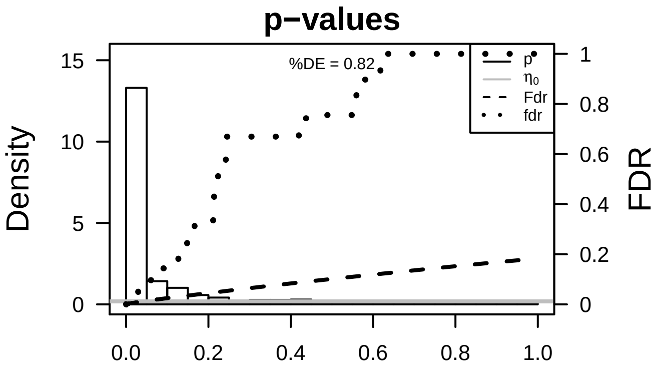

Supplement: Supplementary file 1 [file cells-09-00779-s001.zip › Supplementary materials/FileS4.zip!/FileS3/TFs - Results/Summary Sheets - Differences/Reports/Sprint.2.20..vs.Zhewan.1.25..pdf]
